# Therapeutic Potential of Mesenchymal Stem Cell-Derived Extracellular Vesicles in the Treatment of Parkinson’s Disease

**DOI:** 10.3390/cells14080600

**Published:** 2025-04-16

**Authors:** Ana Volarevic, Carl Randall Harrell, Aleksandar Arsenijevic, Valentin Djonov, Vladislav Volarevic

**Affiliations:** 1 Departments of Psychology, Center for Research on Harmful Effects of Biological and Chemical Hazards, Faculty of Medical Sciences University of Kragujevac, 69 Svetozara Markovica Street, 34000 Kragujevac, Serbia; ana.volarevic@fmn.kg.ac.rs; 2 Regenerative Processing Plant, LLC, 34176 US Highway 19 N, Palm Harbor, FL 34684, USA; 3 Departments of Genetics, Microbiology and Immunology, Center for Research on Harmful Effects of Biological and Chemical Hazards, Faculty of Medical Sciences University of Kragujevac, 69 Svetozara Markovica Street, 34000 Kragujevac, Serbia; aleksandar@medf.kg.ac.rs; 4 Institute of Anatomy, University of Bern, Baltzerstrasse 2, 3012 Bern, Switzerland; valentin.djonov@unibe.ch

**Keywords:** mesenchymal stem cells, extracellular vesicles, Parkinson’s disease, therapy, dopaminergic neurons

## Abstract

Parkinson’s disease (PD) is a progressive neurodegenerative disorder characterized by the gradual loss of dopamine-producing neurons. Oxidative stress, mitochondrial dysfunction, detrimental immune response, and neuroinflammation are mainly responsible for the injury and degeneration of dopaminergic neurons in the brains of patients suffering from PD. Mesenchymal stem cell-derived extracellular vesicles (MSC-EVs) have emerged as a promising therapeutic approach for treating PD due to their ability to suppress the activation of inflammatory immune cells and enhance the viability and function of dopamine-producing neurons. MSC-EVs can easily bypass the blood-brain barrier and deliver their cargo (neuroprotective factors, immunosuppressive proteins, and microRNAs) to injured dopamine-producing neurons and brain-infiltrated inflammatory immune cells. A large number of recently published experimental studies demonstrated that MSC-EVs efficiently alleviated PD-related motor and behavioral deficits in animal models, indicating that MSC-EVs should be considered as potentially new therapeutic agents for the treatment of PD. Accordingly, in this review article, we summarized current knowledge about the therapeutic potential of MSCs-EVs in the treatment of PD, paving the way for their future clinical use in the treatment of neurodegenerative and neuroinflammatory disorders.

## 1. Introduction

Parkinson’s disease (PD) is a progressive neurodegenerative disorder characterized by the presence of Lewy bodies and by the gradual loss of dopamine-producing neurons within the substantia nigra pars compacta (SNpc) of the brain [1]. Lewy bodies are intracellular aggregates primarily composed of alpha-synuclein (αSN), a protein that misfolds and accumulates in dopaminergic neurons, disrupting their function [2]. The loss of dopaminergic neurons leads to decreased levels of dopamine in the striatum [1]. This depletion leads to a range of motor symptoms, including tremors, rigidity, bradykinesia, and postural instability [1]. Gliosis, as a reactive response to neuronal loss, importantly contributes to the development of non-motor symptoms like cognitive decline, sleep disturbances, and mood disorders [1,3].

PD results from a delicate interplay between genetic vulnerabilities and environmental exposures, leading to the development of neurodegeneration and pathological lesions in the brain [3,4,5]. Genetic predisposition plays a significant role, with mutations in protein-coding genes such as Synuclein alpha (SNCA), Leucine-rich repeat kinase 2 (LRRK2), and Parkinsonism-associated deglycase (PARK7) being linked to familial forms of the disease [4,5]. Mutations in the SNCA gene are linked to early-onset forms of the disease and are characterized by abnormal aggregation of αSN, while mutations in the LRRK2 gene are responsible for the development of neuronal toxicity due to the dysfunction of LRRK2 kinase, which modulates the activity of proteins that are involved in dopamine release [3,4]. Other genes, such as PARK7 (DJ-1), Phosphatase and tensin-homolog- (PTEN)-induced kinase 1 (PINK1), and Parkin RBR E3 ubiquitin protein ligase (PRKN), have been implicated in autosomal recessive forms of PD, where mutations in these genes result in the disruption of mitochondrial function and cellular stress responses, contributing to the degeneration of dopaminergic neurons [4,5]. Environmental factors, including exposure to neurotoxins, pesticides, heavy metals, and industrial solvents, have also been implicated in the etiology of PD [1,3]. Aging itself is a significant risk factor, as the incidence of PD increases with age, suggesting that cumulative cellular damage over time may play a critical role in disease onset [1,3].

Oxidative stress, mitochondrial dysfunction, and detrimental immune response crucially contribute to neuroinflammation and neural degeneration in patients with PD [6]. Microglia, resident macrophages in the brain parenchyma, become activated in response to the accumulation of αSN and other misfolded proteins [7]. Upon activation, microglia release pro-inflammatory cytokines (tumor necrosis factor-alpha (TNF-α), interleukin (IL)-1β, and IL-6), which cause oxidative stress in neural cells and create a toxic microenvironment that further contributes to the damage and degeneration of dopaminergic neurons in the SNpc [1,7]. Additionally, microglia-derived TNF-α and IL-1β enhance the expression of E and P selectins and chemokines on endothelial cells (ECs), increasing the permeability of the blood-brain barrier (BBB) [7]. Accordingly, the circulating inflammatory cells, particularly interferon gamma (IFN-γ) and IL-17-producing Th1 and Th17 lymphocytes, can easily bypass BBB and infiltrate the brain of PD patients, exacerbating ongoing neuroinflammation [7,8]. The interplay between activated microglia and recruited immune cells perpetuates a cycle of inflammation and neurodegeneration, which can further hinder neurorestorative processes, such as neurogenesis and synaptic plasticity [9]. Additionally, neuroinflammation can disrupt the delicate balance of neurotransmitter signaling in the brain [9]. The inflammatory cytokines can interfere with dopamine synthesis and release, worsening motor symptoms and contributing to non-motor manifestations such as mood disorders and cognitive decline [1,9]. Therefore, therapeutic strategies targeting neuroinflammation could provide new avenues for slowing progression and improving the quality of life of patients with PD [10].

Current therapeutic approaches for PD primarily focus on managing symptoms [10,11]. The cornerstone of treatment is dopaminergic therapy, with levodopa being the most effective and widely used medication [11]. Once converted to dopamine in the brain, levodopa alleviates motor symptoms significantly, particularly bradykinesia and rigidity. However, prolonged use could lead to motor fluctuations and dyskinesias [3,11]. To mitigate these side effects, clinicians often combine levodopa with carbidopa, a peripheral decarboxylase inhibitor, or add dopamine agonists like pramipexole or ropinirole, which stimulate dopamine receptors directly [3,11]. In addition to pharmacological methods, non-pharmacological strategies, such as physical therapy, occupational therapy, and speech therapy, play crucial roles in maintaining function and mobility [10,12]. Furthermore, there is growing interest in neuroprotective treatments, including inhibitors of mytochondral enzymes like selegiline and rasagiline, which aim to slow disease progression by reducing oxidative stress [10,11]. Innovative approaches, such as gene therapy or the delivery of neuroprotective factors or immunoregulatory proteins in injured neurons and brain-infiltrating immune cells are being explored in recently experimental and clinical trials to target neurodegeneration and neuroinflammation [10,11,12,13,14,15,16,17,18,19,20].

Mesenchymal stem cells (MSCs) are hypoimmunogenic and immunoregulatory adult stem cells that possess a unique array of immunomodulatory properties that make them a compelling option for the treatment of PD [14,15,16,17,18,19,20]. MSCs are considered hypoimmunogenic cells since major histocompatibility class (MHC) II molecules are not highly expressed on their membranes [20]. Harmful allogeneic immune response has not been frequently observed upon the transplantation of MSCs in MHC miss-matched recipients [19,20]. Importantly, MSCs produce various bioactive molecules, immunoregulatory cytokines, and growth factors, which can suppress the progression of detrimental immune responses in the brains of PD patients and may provide trophic support for the repair and regeneration of injured neural tissue [19]. MSCs-derived immunosuppressive factors (IL-10, prostaglandin E2 (PGE2) and transforming growth factor beta (TGF-β) induce the generation of tolerogenic phenotype in dendritic cells (DCs), preventing DC-dependent generation of inflammatory Th1 and Th17 cells in the brain [20]. MSCs-sourced indoleamine 2,3-dioxygenase (IDO) promotes the expansion of immunosuppressive T regulatory cells (Tregs), which inhibit the activation of inflammatory macrophages and attenuate Th1 and Th17 cell-driven neuroinflammation in PD patients [19]. Additionally, MSCs-derived neurotrophic factors (glial cell line-derived neurotrophic factor (GDNF), brain-derived neurotrophic factor (BDNF)) provide protection to injured neurons by enhancing endogenous repair mechanisms and by fostering an environment conducive to the survival and regeneration of neuronal cells [19,20]. In several preclinical and pilot clinical studies, MSCs have demonstrated promising results in improving motor functions and overall quality of life in PD patients, highlighting their ability to repair and regenerate damaged tissues and to suppress detrimental immune responses within the central nervous system [19].

Despite these promising results, there are several safety concerns that limit the clinical use of MSCs in the cell-based therapy of PD [19,21]. One significant challenge is the variability in MSC sources, which can lead to inconsistent therapeutic outcomes. MSCs can be derived from various tissues, including bone marrow (BM), adipose tissue (AT), umbilical cord (UC), dental pulp (DP), and their characteristics can differ significantly based on the donor’s age, health status, and the method of isolation [21]. Uncontrolled proliferation and differentiation of transplanted MSCs represent additional safety concerns of MSC-based therapy [21]. There is also a concern regarding the potential for intravenously infused MSCs to migrate to unintended sites within the body (particularly the lungs and liver), leading to complications that could negate the intended therapeutic effects [21].

MSCs-derived extracellular vesicles (MSC-EVs) effectively address several safety concerns associated with MSC-based therapies, primarily by eliminating many of the risks associated with live cell transplantation [14]. Since MSC-EVs are acellular products, they do not have the capacity for uncontrolled proliferation and differentiation [14,15]. Additionally, MSC-EVs can be engineered to enhance their stability and target specificity, allowing their precise delivery to damaged neural tissues while minimizing systemic exposure and potential side effects [22]. This targeted approach not only enhances safety but also improves therapeutic efficacy, making MSC-EVs a promising alternative in the treatment of neuroinflammatory and neurodegenerative conditions, where effective neuroprotection and anti-inflammatory responses are crucial for patient outcomes [22].

MSC-EVs showed promising therapeutic potential in treating PD by delivering neuroprotective factors, modulating inflammation, and promoting dopaminergic neuron survival, potentially slowing disease progression and improving motor function [14]. MSC-EVs encompass a diverse range of vesicle types, including apoptotic bodies, microvesicles, and exosomes (Exos), each with distinct characteristics and functions [14,15]. Apoptotic bodies are formed during the process of programmed cell death. They are the largest EVs, ranging in size from 1 to 5 μm [15]. Apoptotic bodies are composed of cellular remnants, including fragments of the cytoplasm, organelles, and nuclear material, which are enclosed by a lipid bilayer [14,15]. They typically exhibit a complex composition of proteins, lipids, and nucleic acids, reflecting the cellular context from which they originate [14,15]. Apoptotic bodies can elicit an immune response in the injured or inflamed brain by presenting antigens to the resident immune cells within the brain parenchyma [15]. They also play a role in tissue homeostasis by clearing cellular debris and facilitating repair processes [15]. Microvesicles are EVs that are smaller than apoptotic bodies [14,15]. Their diameter is between 100 and 1000 nanometers [15]. Microvesicles shed directly from the plasma membrane of stem cells and often have a different lipid composition and protein content compared to other EVs, reflecting their origin and role in intercellular communication [15]. Microvesicles play vital roles in mediating neuroinflammation and tissue repair by delivering bioactive molecules to the activated immune cells and injured neurons [15].

Exos are the smallest EVs, typically ranging from 30 to 150 nanometers in diameter, characterized by their spherical shape and a lipid bilayer membrane that encapsulates a complex cargo of proteins, lipids, and nucleic acids [14,15]. Morphologically, they are formed within the endosomal system through the inward budding of the endosomal membrane, resulting in the creation of intraluminal vesicles that accumulate in multivesicular bodies [15]. Upon fusion of these bodies with the plasma membrane, Exos are released into the extracellular environment [15]. Functionally, Exos play a crucial role in intercellular communication, mediating the transfer of stem cell-derived biologically active molecules to recipient cells, influencing immune response, cell proliferation, and differentiation [14,15]. Exos express specific markers such as CD9 CD63, and CD81, which facilitate their uptake by target cells [15]. CD9 facilitates the fusion of MSC-EVs with target cell membranes, enhancing their uptake by recipient cells [14,15]. CD63 is implicated in the endosomal pathways that lead to Exos formation [15]. CD81 is involved in the organization of membrane microdomains, which are essential for the sorting of proteins into Exos [14,15]. Together with integrins, CD81 facilitates the uptake of Exos by target cells through receptor-ligand interactions [14,15]. The protein content of Exos includes a variety of immunoregulatory factors, cytokines, and heat shock proteins, while their lipid composition is rich in ceramides and cholesterol, contributing to their stability and facilitating membrane fusion with target cells [14,15]. Due to their nano-size dimension, lipid bilayer composition, and increased expression of surface proteins that facilitate receptor-mediated endocytosis, Exos are able to easily bypass the BBB, allowing targeted delivery of their content directly to injured neurons and brain-infiltrated immune cells [14]. Importantly, MSC-EVs are enriched with different microRNAs (miRNAs), which can modulate gene expression in target cells, highlighting their therapeutic potential in the treatment of neuroinflammatory and neurodegenerative diseases, including PD [14,15].

The results obtained in several recently published studies demonstrated that, similar to MSC-EVs, neural stem cells (NSCs) and cerebral organoids-sourced EVs can successfully attenuate PD in experimental animals [16,17,18]. However, the therapeutic application of these EVs encountered several notable obstacles [16,17,18]. Their yield and composition can differ significantly based on the source and culture conditions of their parental cells, potentially leading to inconsistent therapeutic effects. Additionally, there is a serious concern regarding the potential immunogenicity of these EVs, which could trigger detrimental and life-threatening immune responses in EVs-treated patients [16,17,18].

In contrast to NSCs and organoids-sourced EVs, MSC-EVs have emerged as a promising therapeutic approach for treating PD due to their ability to promote the healing of injured neurons in an immunoprivileged manner [22]. In numerous preclinical studies, MSC-EVs have demonstrated the capacity to suppress detrimental immune responses in the brain by inhibiting the activation of microglia and lowering pro-inflammatory cytokine levels, effectively countering the detrimental effects of neuroinflammation [23,24,25]. Additionally, MSC-EVs are able to facilitate synaptic repair and neurogenesis, leading to improved motor function and cognitive outcomes in animal models of PD [26,27]. Their ability to cross the BBB and deliver their cargo directly to injured neurons and inflammatory immune cells further enhances their therapeutic potential [26,27]. Accordingly, in this review article, we summarized current knowledge about the therapeutic potential of MSCs-EVs in the treatment of PD. An extensive literature review was carried out in January 2025 across several databases (MEDLINE, EMBASE, and Google Scholar), from 1990 to the present. Keywords used in the selection were: “mesenchymal stem cell-derived extracellular vesicles”, “Parkinson’s disease”, “neurodegeneration”, “neuroinflammation”, “targeted therapy”, “signaling pathways”, “growth factors”, and “tissue repair and regeneration”. All journals were considered and an initial search retrieved 137 articles. The abstracts of all these articles were subsequently reviewed by two of the authors (AV and CRH) independently to check their relevance to the subject of this manuscript. Eligible studies had to delineate the molecular and cellular mechanisms that are involved in MSC-EVs-dependent attenuation of neurodegeneration and neuroinflammation in PD, and their findings are analyzed in this review.

## 2. Therapeutic Potential of MSC-EVs in the Treatment of PD: What Have We Learnt from In Vivo Models?

**Figure 1:**
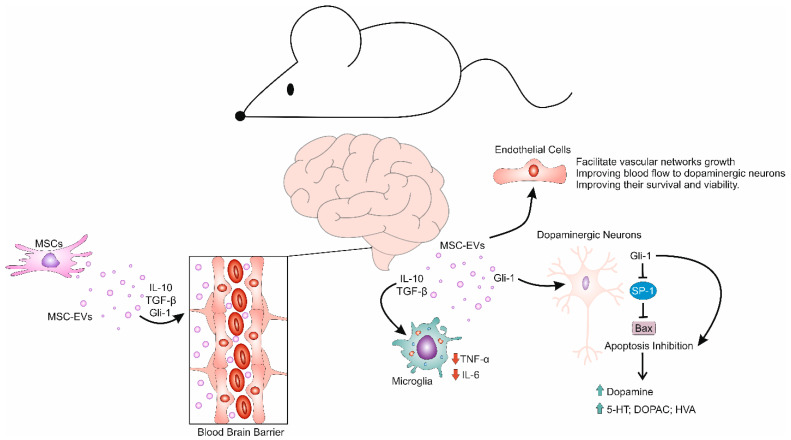
Molecular mechanisms responsible for MSC-EVs-dependent recovery of injured dopaminergic neurons. MSC-EVs easily bypass the BBB and deliver various neurotrophic and immunosuppressive factors to injured dopaminergic neurons, enhancing their viability and function. Additionally, MSC-EVs attenuate microglia-driven inflammatory responses and prevent inflammation-induced injury of dopaminergic neurons. By delivering IL-10 and TGF-β, MSC-EVs suppress the production of inflammatory cytokines (TNF-α and IL-6) in microglia and increase the production of dopamine, 5-hydroxytryptamine (5-HT) and their metabolites (3,4-Dihydroxyphenyl acetic acid (DOPAC), and homovanillic acid (HVA)) in injured dopaminergic neurons. Additionally, MSC-EVs-sourced Gli-1 prevents apoptosis of dopaminergic neurons by suppressing the activation of specificity protein 1 (SP-1), which enhances the expression of the pro-apoptotic Bax gene. Additionally, by activating SMAD3 and p38/MAPK signaling pathways in brain endothelial cells, MSC-EVs facilitates the growth of vascular networks, which improves blood flow to injured dopaminergic neurons, improving their survival and viability.

The results recently published in a large number of experimental studies indicated that MSC-EVs should be considered as potentially novel therapeutic agents in the treatment of PD [28,29,30,31,32,33,34,35,36]. Huang and colleagues demonstrated that umbilical cord-derived MSC-EVs (UC-MSC-EVs) efficiently suppressed microglia-driven neuroinflammation, supported the survival of injured dopaminergic neurons, improved behavioral functions, and prevented the progression of PD in experimental mice [Figure 1] [28]. Upon their intranasal administration, UC-MSC-EVs easily crossed the BBB and mainly accumulated in the SNpc. These EVs were endocytosed by both olfactory and dopaminergic neurons [28]. UC-MSC-EVs increased the activity of olfactory bulb neurons and reversed dopaminergic neuron loss in the SNpc. Additionally, UC-MSC-EVs were also accumulated within astrocytes and microglia, affecting their phenotype and function [28]. UC-MSC-EVs inhibited the activation of astrocytes and microglia, suppressed the production of inflammatory cytokines, attenuated microglia-driven inflammatory responses, and prevented inflammation-induced injury of dopaminergic neurons [28]. Importantly, significantly improved motor and non-motor functions were observed in UC-MSC-EVs-treated PD mice, confirming the beneficial effects of UC-MSC-EVs-dependent immunosuppression and neuroprotection in PD treatment [28]. However, despite these promising expectations, several limitations of this study should be emphasized before UC-MSC-EVs can be offered as potential therapeutic agents for the treatment of PD in clinical settings. Although Huang and colleagues described in detail the molecular mechanisms responsible for UC-MSC-EVs-based modulation of microglia-driven inflammation, the effects of these EVs on adaptive immunity in the inflamed brains of PD mice were not analyzed in this study [28]. The crosstalk between inflammatory T cells and microglia plays a crucially important role in the development and progression of PD [7,8]. Accordingly, to confirm the main conclusions presented by Huang and colleagues, future experimental studies should concomitantly evaluate the effects of UC-MSC-EVs on T cell and microglia-driven immune responses in the brains of PD animals [28].

In line with these observations are results obtained by Zhang et al., who described the efficacy of intravenously injected UC-MSC-EVs, which successfully bypassed the BBB and accumulated in dopaminergic neurons and microglia in 6-Hydroxydopamine (6-OHDA)-treated PD rats [29]. UC-MSC-EVs suppressed the activation of microglia and diminished the production of inflammatory cytokines (IL-1β and IL-18) in the brains of experimental animals [28]. Accordingly, inflammation-induced injury of dopaminergic neurons was inhibited, and PD-related pathological changes, such as shrunken neurons, the unclear boundary between the nucleus and cytoplasm, and the lost interstitial space between neurons in the SNpc, were not observed in the 6-OHDA+UC-MSC-EVs-treated rats [28]. UC-MSC-EVs improved the viability of dopaminergic neurons, increased the production of dopamine, 5-hydroxytryptamine (5-HT), and their metabolites (3,4-Dihydroxyphenyl acetic acid (DOPAC) and homovanillic acid (HVA)), and enhanced the recovery of motor functions in experimental animals [28]. The generalizability of these findings could be limited by the fact that the impact of UC-MSC-EVs on the pathological accumulation of αSN in the brains of experimental animals, was not analyzed [28].

In line with these observations, Aliakbari and colleagues conducted an experimental study to analyze the beneficial effects of UC-MSC-EVs loaded with baicalein and oleuropein, hydrophobic small molecules that are able to inhibit αSN fibrillation and aggregation in dopaminergic neurons [30]. Since zwitterionic nanoliposomes (NLPs) stabilize baicalein and maintain its antioxidant and neuroprotective activity, Aliakbari et al. developed UC-MSC-EVs:NLP hybrids and used them as vehicles for targeted delivery of baicalein (UC-MSC-EVs-NLP-Ba) and oleuropein (UC-MSC-EVs-NLP-Ole) [30]. UC-MSC-EVs-NLP-Ba and UC-MSC-EVs-NLP-Ole hybrids prevented αSN fibrillation in dopaminergic neurons [30]. Importantly, UC-MSC-EVs improved the capacity of NLP-Ba and NLP-Ole to bypass the BBB and significantly enhanced their capacity to reduce αSN-dependent neurotoxicity [30]. Based on these findings, the long-term neuroprotective effects of UC-MSC-EVs-NLP-Ba and UC-MSC-EVs-NLP-Ole hybrids should be confirmed in upcoming experimental and clinical studies.

By using the 6-OHDA-model of PD, Chen and colleagues explored the potential of intravenously infused UC-MSC-EVs to restore dopamine production in injured SNpc-residing neurons [31]. They found that UC-MSC-EVs alleviated apomorphine-induced asymmetric rotations and minimized apoptotic cell loss among dopaminergic neurons [31]. Moreover, signs of degeneration and necrosis, characterized by deeply eosinophilic cytoplasm, pyknosis, and karyolysis, were absent in the SNpc of 6-OHDA+UC-MSC-EVs-treated rats [31]. Histological examinations demonstrated marked improvements in brain tissue from these animals, including the presence of healthy multipolar neurons with nucleoli and basophilic granular cytoplasm [31]. Importantly, UC-MSC-EVs significantly raised levels of dopamine and its metabolites (DOPAC and HVA) in the striatum, indicating that UC-MSC-EVs managed to restore the function of injured dopaminergic neurons in 6-OHDA rats [31]. Future experimental studies should delineate the exact signaling pathways that were altered in the dopaminergic neurons of UC-MSC-EVs-treated animals, enabling complete restoration of dopamine production in the central nervous system.

By using α-SN A53T transgenic mice, an animal model of progressive PD, Xu and colleagues demonstrated that BM-derived MSC-EVs (BM-MSC-EVs) could significantly improve the motor, learning, and memory ability of PD mice by affecting phospholipid composition and cholesterol metabolism in hippocampal neurons [32]. Although cholesterol is essential for the formation of lipid rafts, which facilitate synaptic function and neurotransmitter release, its dysregulated metabolism can lead to the accumulation of neurotoxic metabolites such as 24S-hydroxycholesterol and 27-hydroxycholesterol [33]. These oxidized derivatives can disrupt neuronal function by inducing oxidative stress, promoting inflammation, and impairing mitochondrial function, all of which are detrimental to dopaminergic neurons [33]. Intrastrial injection of BM-MSC-EVs decreased the levels of 24S-hydroxycholesterol and increased total cholesterol levels in the brains of α-SN A53T transgenic mice [32]. Liquid chromatography-mass spectrometry revealed that BM-MSC-EVs modulated the metabolism of brain cholesterol by down-regulating relative percentages of phosphatidylglycerol and phosphatidylethanolamine and by up-regulating phosphatidylinositol, phosphatidylserine, and phosphatidylcholine [32]. The generalizability of these findings may be limited by the fact that BM-MSC-EVs-dependent effects on brain-infiltrated immune cells were not analyzed in this study.

The therapeutic potential of BM-MSC-EVs was demonstrated by Cai and colleagues, who showed that BM-MSC-EVs efficiently suppressed microglia-driven neuroinflammation and attenuated PD progression in experimental animals [34]. By delivering Glioma-associated oncogene homolog 1 (Gli-1) in microglia, BM-MSC-EVs reprogramed inflammatory microglia towards an immunosuppressive phenotype, promoted the transcription of anti-inflammatory mediators IL-10 and TGF-β, dampened TNF-α and IL-6-driven injury of dopaminergic neurons, and alleviated microglia-driven neuroinflammation in the SNpc [34]. In addition to its anti-inflammatory effects, BM-MSC-EVs-sourced Gli-1 prevented apoptosis of dopaminergic neurons by suppressing the activation of specificity protein 1 (SP-1), which is up-regulated in response to oxidative stress and neuroinflammation. SP-1 overexpression leads to the transcription of the pro-apoptotic Bax gene, resulting in the apoptosis of dopaminergic neurons [34]. By delivering Gli-1, BM-MSC-EVs increased the viability of dopaminergic neurons, making them less vulnerable to SP-1-induced apoptosis [34]. The long-term effects of BM-MSC-EVs-sourced Gli-1 on the restoration of PD-related symptoms were not analyzed in this study and should be explored in upcoming experimental studies [34].

MSC-EVs were also able to efficiently attenuate sleep disorder, one of the most common non-motor symptoms in PD [35]. Intravenously injected MSC-EVs accumulated in the brains of 6-OHDA-treated animals and stimulated the production of dopamine and 5-HT. MSC-EVs induced increased synthesis of Brain and muscle arnt-like protein-1 (BMAL1) and Period circadian regulator 2 (PER2) proteins, which are integral components of the circadian clock that regulate circadian rhythm, metabolism, and the viability of injured neurons [35]. Additionally, MSC-EVs restored mitochondrial membrane potential in dopaminergic neurons and promoted the activation of peroxisome proliferator-activated receptor gamma (PPARγ), which, as a nuclear receptor, regulates the expression of genes that are involved in lipid metabolism, glucose homeostasis, and oxidative stress-induced injury [35]. Total sleep time, slow-wave sleep time, and fast-wave sleep time were prolonged in 6-OHDA+MSC-EVs-treated rats, indicating that MSC-EVs managed to significantly improve sleep disorder in PD rats through recovering circadian rhythm-associated gene expression [35]. Despite these promising results, it should be noted that in this study, Li et al. did not investigate the long-term effects of MSC-EVs on sleep disorders in PD animals, leaving open questions about the durability of the observed improvements and their implications in clinical settings.

Neo-angiogenesis offers promising therapeutic benefits in the treatment of PD by enhancing cerebral perfusion and promoting neuroprotection [11]. In response to the neurodegenerative processes, the recruitment of new blood vessels can facilitate the delivery of essential nutrients and oxygen into the SNpc, supporting the survival of dopaminergic neurons [11]. Additionally, angiogenesis can improve the removal of toxic metabolic waste products, which is crucial for maintaining a healthy microenvironment in the brain [1,3]. In line with these findings, Xue and colleagues showed that MSC-EVs promoted the generation of new blood vessels in the SNpc of PD mice by activating Suppressor of mothers against decapentaplegic 3 (SMAD3) and p38/Mitogen-activated protein kinase (MAPK) signaling pathways in brain ECs [36]. Through the activation of the SMAD3 protein, which promotes EC proliferation and migration, MSC-EVs facilitated the growth of vascular networks, which improved blood flow to degenerated neurons. Concurrently, MSC-EVs-dependent activation of the p38/MAPK pathway in ECs promoted the secretion of pro-angiogenic factors and contributed to the enhanced survival of endothelial cells under neurotoxic conditions, alleviating the progression of PD in experimental animals [36]. The main limitation of this study is that it did not examine the potential adverse effects of enhanced neo-angiogenesis induced by MSC-EVs, such as the risk of abnormal blood vessel formation or increased inflammation, which could counteract the intended neuroprotective benefits in PD [36].

## 3. Molecular Mechanisms Responsible for the Beneficial Effects of MSC-EVs in the Treatment of PD

Neuroinflammation, which is present in the brains of PD patients, significantly increases the permeability of the BBB, enabling enhanced passage of MSC-EVs [10,24]. Additionally, MSC-EVs express various chemokine receptors (C-C chemokine receptor (CCR)1, CCR2, CCR4, and C-X-C chemokine receptor (CXCR)4), which bind to chemokine C-C motif ligand (CCL)-2 and C-X-C motif ligand (CXCL)12, both of which are highly expressed in the ECs of the inflamed brains of PD patients [22,23]. Accordingly, MSC-EVs may respond to these inflammatory chemokines and migrate toward inflamed tissues, effectively delivering therapeutic molecules in injured neurons and brain-infiltrated immune cells [22,23]. MSC-EVs are enriched with MSC-sourced neuroprotective and immunoregulatory proteins, which may attenuate the progression of neurodegeneration and neuroinflammation in the brains of PD patients [Table 1] [15]. Precisely, MSC-EVs contain MSC-sourced BDNF, GDNF, and nerve growth factor (NGF), all of which support and promote the growth and survival of dopaminergic neurons [23,24,25]. MSC-EVs also contain the enzymes superoxide dismutase (SOD) and catalase, which combat oxidative stress by neutralizing reactive oxygen species (ROS), ultimately safeguarding dopaminergic neurons from damage [24,25]. Additionally, MSC-EVs carry neuroprotective miRNAs (miR-133b, miR-21, and miR-124) that regulate gene expression, protect against apoptosis, and promote resilience of dopaminergic neurons [Table 2] [22,23,24]. Collectively, these MSC-derived bioactive molecules work synergistically to create a protective milieu that could slow down the progression of PD by preserving neuronal integrity, promoting cell survival, and reducing oxidative damage [22,23,24,25].

In addition to neuroprotective proteins and miRNAs, MSC-EVs also contain various immunomodulatory proteins (interleukin 1 receptor antagonist (IL-1Ra}, hepatocyte growth factor (HGF), IL-10, TGF-β, IDO, TNF-stimulated gene-6 (TSG-6)), which could efficiently suppress detrimental immune responses in the brains of PD patients, alleviating ongoing neuroinflammation [22,23]. MSC-sourced IL-1Ra is a naturally occurring antagonist of the pro-inflammatory cytokine IL-1β, which is often up-regulated in PD and associated with neuroinflammation [22,23]. By binding to IL-1 receptors, IL-1Ra effectively blocks the IL-1β-driven inflammatory signaling pathway that leads to further neuronal damage [3]. This inhibition helps to reduce the recruitment of inflammatory cells to the brain, thus lowering the overall inflammatory burden [22,23]. MSC-derived IL-10 is a potent anti-inflammatory cytokine that can efficiently suppress ongoing inflammatory responses in the brains of PD patients [22,23]. It suppresses the production of pro-inflammatory cytokines (TNF-α, IL-1β, and IL-6) in macrophages and neutrophils, thereby fostering an environment conducive to neuronal survival [23]. Additionally, MSC-sourced IL-10 promotes the polarization of microglia towards a neuroprotective phenotype, which facilitates tissue repair and reduces the release of harmful mediators [23]. Similarly, MSC-derived IL-10 and TGF-β induce the generation of tolerogenic phenotype in DCs and promote alternative activation of macrophages, enabling the creation of an immunosuppressive environment in inflamed brains that may efficiently protect dopaminergic neurons [22,23]. Furthermore, MSC-EVs contain IDO, which promotes the expansion of Tregs that can, via a juxtacrine and paracrine manner, suppress the activation and proliferation of inflammatory Th1 and Th17 lymphocytes, attenuating T cell-driven inflammation in the brains of PD patients [23]. MSC-EVs are also enriched with neuroprotective TSG-6 and HGF [23,24,37]. MSC-EVs-derived TSG-6 efficiently reduces oxidative stress and attenuates 1-methyl-4-phenylpyridinium (MPP+)-induced neurotoxicity by modulating signal transducer and activator of transcription 3 (STAT3)/miR-7/LRKK2-driven signaling pathway [37]. MSC-sourced HGF supports the survival and promotes the recovery of dopaminergic neurons by suppressing the activation of microglia, making MSC-EVs a valuable therapeutic candidate for mitigating the neuroinflammatory aspects of PD [23,24].

**Figure 2:**
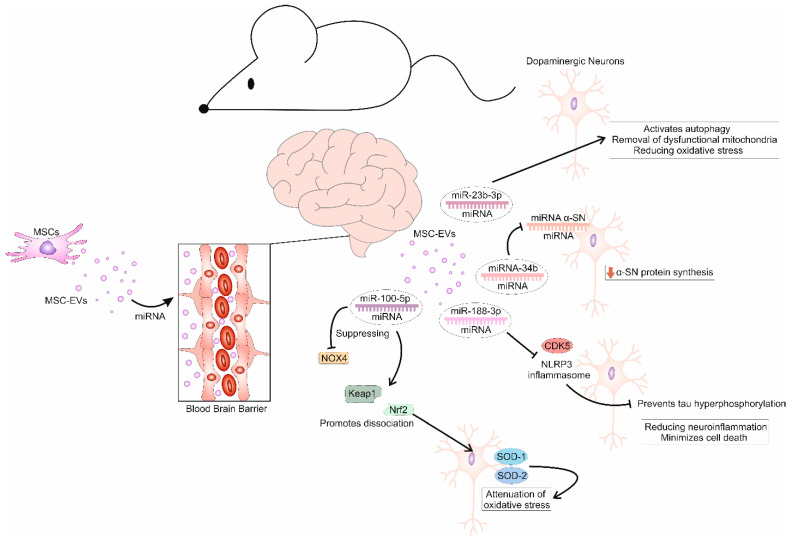
Beneficial effects of MSC-EVs-sourced miRNAs in the attenuation of PD. MSC-EVs carry neuroprotective and immunoregulatory miRNAs that regulate gene expression, protect against apoptosis, and promote the resilience of dopaminergic neurons. MSC-EVs-derived miR-100-5p suppresses NOX4 activity by promoting the dissociation of Nrf2 from Keap1. Subsequently, Nrf2 translocates to the nucleus and activates the expression of antioxidant enzymes SOD-1 and SOD-2, thereby attenuating oxidative stress in dopaminergic neurons. MSC-EVs-derived miRNA-34b targets α-SN mRNA, causing a decrease in α-SN protein synthesis. MSC-EVs-sourced miR-23b-3p activates autophagy and supports mitochondrial health by facilitating the removal of dysfunctional mitochondria, thereby reducing oxidative stress in dopaminergic neurons. By suppressing the activation of CDK5 and the NLRP3 inflammasome, MSC-EVs-sourced miR-188-3p prevents tau hyperphosphorylation, reduces neuroinflammation, and minimizes cell death of dopaminergic neurons, thereby attenuating the progression of PD in experimental animals.

Numerous studies have demonstrated that MSC-EVs-derived miRNAs are mainly responsible for the beneficial effects of MSC-EVs in the alleviation of PD [Figure 2] [38,39,40,41,42]. He and colleagues used a murine model of 1-methyl-4-phenyl-1,2,3,6-tetrahydropyridine (MPTP)-induced PD to evaluate the therapeutic potential of EVs obtained from trophoblast stage-derived MSCs (T-MSCs-EVs) [38]. Intravenously injected T-MSCs-EVs successfully crossed the BBB and accumulated within the SNpc, where they were taken up by dopaminergic neurons via endocytosis [38]. T-MSCs-EVs significantly enhanced the viability and improved the functionality of these neurons, resulting in improved motor function and behavioral performance in MPTP-injured mice [38]. Interestingly, behavioral tests showed that the effectiveness of T-MSC-EVs depended on their dose. The best therapeutic effects were achieved when T-MSCs-Exo were given at a dose of 7.32 × 10^10^ particles/mL every 14 days (on days 3, 17, and 31) [38]. Sequencing analysis showed that among the large number of MSC-derived miRNAs, miR-100-5p, miR-21-5p, miR-320a-3p, miR-221-3p, miR-146a-5p, miR-335-5p, miR-423-5p, miR-191-5p, miR-92a-3p, and miR-30e-5p were the most abundantly present in T-MSC-EVs [38]. Bioinformatical analysis revealed that miR-100-5p-dependent modulation of the Nicotinamide adenine dinucleotide phosphate hydrogen oxidase 4 (NOX4) gene was mainly responsible for the beneficial effects of T-MSCs-EVs in the attenuation of neural degeneration [38]. In PD, NOX4 activation leads to the accumulation of ROS, inducing oxidative stress and damage to dopaminergic neurons [5,6]. Additionally, NOX4-driven oxidative stress disrupts mitochondrial function, triggers apoptosis, and promotes neuroinflammation [6]. Accordingly, targeting NOX4 may offer therapeutic potential by reducing oxidative stress and protecting against dopaminergic neuronal loss [11]. He and colleagues showed that T-MSCs-EVs-derived miR-100-5p down-regulated NOX4 expression by directly targeting the 3′ UTR of NOX4 [38]. By down-regulating NOX4 activity, T-MSC-EVs-derived miR-100-5p promoted the dissociation of nuclear factor erythroid 2-related factor 2 (Nrf2) from Kelch-like ECH-associated protein 1 (Keap1). Nrf2 was then translocated to the nucleus and activated the expression of antioxidant enzymes SOD-1 and SOD-2, attenuating oxidative stress in dopaminergic neurons [38]. In this way, T-MSCs-EVs-derived miR-100-5p protected against the loss of dopaminergic neurons, supported the functionality of the nigro-striatal system, and significantly improved motor deficits in PD mice, demonstrating the therapeutic potential of T-MSCs-EVs in PD treatment [38].

It is well known that the activation of the Wingless (Wnt)/β-catenin signaling pathway can promote autophagy in dopaminergic neurons, which may be highly beneficial in attenuating the progression of PD [42]. In line with these findings, Geng and colleagues used an animal model of 6-OHDA-induced PD to demonstrate that EVs derived from AT-sourced MSCs (AT-MSC-EVs) contain miR-23b-3p, which could efficiently alleviate PD progression by inducing autophagy in dopaminergic neurons through the activation of the Wnt/β-catenin signaling pathway [39]. AT-MSC-EVs-derived miR-23b-3p promoted autophagy in dopaminergic neurons, as evidenced by elevated levels of p-β-catenin, dopamine transporter (DAT), microtubule-associated protein light chain 3 (LC3BII/I), and Beclin-1 in 6-OHDA+AT-MSC-EVs-treated rats [39]. By enhancing autophagy, AT-MSC-EVs-derived miR-23b-3p promoted the clearance of damaged proteins and organelles, including α-SN [39]. By inhibiting the aggregation of α-SN, AT-MSC-EVs maintained cellular homeostasis and prevented the degeneration of dopaminergic neurons in the brains of PD rats [39]. Additionally, miR-23b-3p-dependent activation of autophagy supported mitochondrial health by facilitating the removal of dysfunctional mitochondria, thereby reducing oxidative stress in dopaminergic neurons [39]. Accordingly, AT-MSC-EVs-based treatment improved neuronal survival and function, ultimately alleviating motor symptoms in experimental animals [39]. Importantly, when ICG-001, an inhibitor of the Wnt pathway, was given to AT-MSC-EVs+6-OHDA-treated rats, all miR-23b-3p-dependent beneficial effects were diminished, emphasizing the crucially important role of Wnt-driven signaling for miR-23b-3p-dependent activation of autophagy in dopaminergic neurons [39].

By using a murine model of MPTP-induced PD, Li and colleagues demonstrated that the beneficial effects of AT-MSC-EVs mainly relied on miR-188-3p-dependent suppression of Nacht Leucine-rich repeat Protein 3 (NLRP3) inflammasome-driven neuroinflammation and cell division protein kinase 5 (CDK5)-dependent pyroptosis of dopaminergic neurons [40]. CDK5 and NLRP3 inflammasomes become activated in response to mitochondrial dysfunction and the accumulation of neurotoxic proteins [1,3]. Activation of CDK5 can lead to hyperphosphorylation of tau proteins and disruption of neuronal integrity, enhancing vulnerability of dopaminergic neurons to pyroptotic cell death [1,3]. Upon activation, the NLRP3 inflammasome induces enhanced production of inflammatory cytokines, IL-1β and IL-18, in microglia, exacerbating damage of dopaminergic neurons in the SNpc [10]. Together, CDK5 and NLRP3 inflammasome-driven cascades create a neurotoxic environment that accelerates dopaminergic neuron degeneration, significantly contributing to the progression of PD [3,10]. Li and colleagues showed that AT-MSC-EVs-derived miR-188-3p targeted specific regions within the mRNA of CDK5 and NLRP3, leading to translational repression and degradation of these mRNAs [40]. By suppressing the activation of CDK5, AT-MSC-EVs-sourced miR-188-3p prevented tau hyperphosphorylation in dopaminergic neurons [40]. Similarly, by targeting NLRP3, AT-MSC-derived miR-188-3p inhibited the activation of the inflammasome in microglia, alleviating the production of inflammatory cytokines IL-1β and IL-18 [40]. Accordingly, miR-188-3p-dependent dual targeting of CDK5 and NLRP3-driven signaling cascades reduced neuroinflammation and minimized cell death of dopaminergic neurons, attenuating the progression of PD in experimental animals [40].

Despite its side effects and lack of efficacy for non-dopaminergic symptoms, levodopa has potent antioxidant properties and, therefore, represents one of the most commonly used drugs for PD treatment [11]. Accordingly, Mohamed et al. recently used a rat model of PD to compare the effects of levodopa with the effects of MSC-EVs [41]. MSC-EVs, which were intravenously administered once a week for three consecutive weeks, successfully inhibited the aggregation of α-SN and, more efficiently than levodopa, improved the motor and cognitive function of PD rats [41]. The therapeutic effects of MSC-EVs were attributed to their capacity to down-regulate the expression of circular RNA (circRNA).2837 and to increase the expression of miR-34b in the brains of experimental animals [41]. MiR-34b targets the 3′-UTR of α-SN mRNA, causing a decrease in α-SN protein synthesis [41]. On the contrary, circRNA.2837 stimulates the aggregation of α-SN protein. CircRNA.2837 binds to miRNA-34b and acts as a “sponge” that removes miRNA-34b, alleviating its capacity to reduce the accumulation of α-SN protein [41]. Therefore, increased levels of circRNA.2837 and the reduced presence of miRNA-34b lead to the formation of α-SN aggregates in the SNpc of PD patients, resulting in bradykinesia and postural instability [1,3]. As evidenced by Mohamed et al., MSC-EVs managed to inhibit the circRNA.2837-dependent suppression of miRNA-34b, enabling the restoration of motor functions in MSC-EVs-treated PD rats [41].

## 4. MSC-EVs as Nano-Sized Vehicles for Targeted Delivery of Bioactive Molecules into Dopaminergic Neurons

The beneficial effects of BDNF in the attenuation of PD are well known [43]. BDNF protects dopaminergic neurons from oxidative stress-induced injury, supports synaptic plasticity, improves learning and memory, and enhances dopamine production by promoting the synthesis of tyrosine hydroxylase (TH), which is responsible for converting the amino acid tyrosine into levodopa, the precursor of dopamine [43]. Based on these findings, Wang and colleagues loaded BDNF in MSC-EVs (MSC-EVs^BDNF^) and used these EVs for targeted delivery of BDNF in injured dopaminergic neurons [44]. After their intravenous administration, MSC-EVs^BDNF^ successfully bypassed the BBB and accumulated within injured dopaminergic neurons, significantly improving their survival [44]. MSC-EVs^BDNF^ facilitated the remodeling of synaptic connections and promoted the brain’s ability to adapt and maintain function despite significant neuronal loss [44]. MSC-EVs^BDNF^ enhanced the stability of the neuronal cytoskeleton by affecting the synthesis and posttranslational modifications of microtubule-associated protein 2 (MAP2) and phosphorylated tau protein (p-tau) [44]. MSC-EVs^BDNF^ enhanced the expression of MAP2, which played a critical role in maintaining the structural integrity and stability of neuronal microtubules. Additionally, MSC-EVs^BDNF^ inhibited the abnormal accumulation of p-tau, preventing the formation of neurofibrillary tangles that can disrupt synaptic function and exacerbate the progression of PD [44]. Additionally, MSC-EVs^BDNF^ strengthened cellular antioxidant defenses in dopaminergic neurons by activating the Nrf2 signaling pathway, which serves as a crucial cellular defense mechanism against oxidative stress. By activating Nrf2, MSC-EVs^BDNF^ supported mitochondrial function and enhanced the overall resilience of dopaminergic neurons in the brains of MSC-EVs^BDNF^-treated PD mice, offering a potentially new therapeutic option for PD treatment [44].

N6-methyladenosine (m6A) modification is a crucial post-transcriptional modification that affects the stability, translation, and splicing of RNA [45]. Changes in m6A levels can affect the translation of mRNA encoding αSN, potentially influencing its production and aggregation [45]. This interaction may contribute to the pathogenesis of PD by promoting the toxic effects of aggregated αSN [45]. Precisely, a decrease in m6A levels in the striatum region leads to a notable reduction in dopamine production [45]. Given that dysregulation of m6A modification contributes to the pathogenesis of PD, Geng and colleagues encapsulated small interfering RNA (siRNA) designed to specifically target and silence the Fat mass and obesity-related (FTO) gene in MSC-EVs, creating MSC-EVs-si-FTO [46]. In this way, MSC-EVs delivered si-FTO into the dopaminergic neurons and prevented the synthesis of FTO protein—an RNA demethylase involved in the regulation of m6A modifications on mRNA [45]. By attenuating FTO production, MSC-EVs-si-FTO reduced the increase of αSN and prevented the decrease of TH, enabling increased production of dopamine [46]. Additionally, MSC-EVs-mediated delivery of si-FTO prevented programmed cell death of dopaminergic neurons, alleviating PD progression in experimental mice [46].

## 5. The Main Benefits and Challenges of Utilizing MSC-EVs in Clinical Practice

MSC-EVs have emerged as compelling therapeutic agents in clinical settings of regenerative neurology, particularly due to their ability to mediate various biological processes in the inflamed and injured central nervous system without the complexities and risks associated with live cell-based therapies [14,15]. MSC-EVs are able to successfully promote tissue repair, modulate immune responses, and reduce inflammation, thereby enhancing recovery outcomes in patients suffering from neuroinflammatory and neurodegenerative diseases [14]. As our understanding of MSC-EVs advances, they hold the promise of becoming a transformative tool in clinical practice, offering a novel approach to treating a variety of conditions, minimizing the risks associated with traditional MSC-based therapies [14].

One of the primary advantages of using MSC-EVs in clinical practice is their inherent ability to mimic the therapeutic properties of their parental MSCs while mitigating many risks associated with the administration of MSCs [14,15,24,25]. The small size of MSC-EVs allows them to effectively cross the BBB, enhancing their therapeutic potential in treating PD and other neurological disorders [15,24]. MSC-EVs can be produced in a controlled and scalable manner, making them a more feasible option for widespread clinical use [15]. Their cargo can be tailored to enhance specific therapeutic effects, and advances in biomanufacturing techniques enable the generation of MSC-EVs with optimized properties for targeted therapies [15].

Standardized methods for the isolation and characterization of MSC-EVs are essential to ensure consistency, reproducibility, and therapeutic efficacy [15]. First, the starting material, whether it be cell culture supernatant or biological fluids, should be standardized, including the source of MSCs, culture conditions, and media composition to reduce variability [14,15]. Isolation techniques such as ultracentrifugation, size exclusion chromatography (SEC), or commercial isolation kits must be clearly defined methods and validated for yield and purity [15]. The characterization of EVs should adhere to specific criteria, including the assessment of size, concentration, and morphology, using techniques like nanoparticle tracking analysis or electron microscopy [15]. For MSC-EVs, the presence of specific markers (CD9, CD63, CD81) on their membranes must be confirmed [15]. Furthermore, molecular profiling of MSC-EVs-containing proteins, lipids, and nucleic acids should be conducted to evaluate functional components relevant to therapeutic mechanisms [14,15]. Quality control measures must also be implemented, including testing for contaminants and ensuring batch-to-batch consistency [15]. Establishing these standardized methods will facilitate the translation of MSC-derived EVs into clinical applications and promote regulatory compliance [14,15].

Another significant hurdle is the variability in MSC-EV production, which can be influenced by factors such as the source of their parental MSCs, the method of isolation, and the culture conditions. This variability can lead to inconsistent therapeutic outcomes [15,22,23]. AT-MSC-EVs and EVs derived from amniotic fluid-sourced MSCs (AF-MSC-EVs) have high proliferative capacity and the ability to secrete various neurotrophic factors that may efficiently support the survival, repair, and regeneration of injured dopamine-producing neurons [22,23,24,25]. BM-MSCs and UC-MSCs possess robust immunosuppressive properties and could be used for the isolation of EVs, which can most effectively suppress ongoing neuroinflammation [22,23,24,25]. The specific microenvironment and the unique biochemical signals present in each tissue source can thus shape the MSCs’ functional abilities, influencing their efficacy in alleviating PD symptoms and promoting neuroprotection, ultimately highlighting the importance of selecting the appropriate MSC source for optimal therapeutic outcomes of their EVs [22,23,24,25]. Therefore, accurately characterizing the exact molecular content of MSC-EVs is crucial for ensuring their effectiveness [47,48,49].

The isolation and purification of MSC-EVs from biological fluids require adherence to specific guidelines to enhance efficiency and purity [15,49]. It is crucial to standardize the collection process of biological fluids to minimize variability, ensuring consistent starting material for the isolation of MSC-EVs [15,47,49]. While traditional methods like ultracentrifugation are widely used, they need to be optimized to reduce processing time and enhance separation efficacy, possibly by incorporating gradient centrifugation techniques to improve purity. Newer approaches such as SEC should be explored further, focusing on enhancing scalability and throughput to meet the demands of clinical applications [15,47,49]. Additionally, integrative techniques combining multiple methods, such as combining SEC with precipitation or filtration, could help maximize yield and purity. However, challenges remain regarding the identification and removal of contaminants, including proteins and lipids that could affect the functionality of obtained MSC-EVs. There is also a need for robust characterization protocols to ensure the identity and biological activity of isolated MSC-EVs, as inconsistencies in MSC-EVs’ quality can significantly impact therapeutic outcomes [15,47,49].

Finally, regulatory considerations should not be disregarded. Regulatory agencies like the Food and Drug Administration (FDA) necessitate detailed manufacturing processes and safety evaluations for MSC-based products [15,47,49]. Establishing specific guidelines for these therapeutics is vital for their successful transition from research to clinical application, addressing concerns related to immunogenicity, stability, and long-term patient outcomes [15,47,49]. First, comprehensive documentation of manufacturing processes, including standardized protocols for cell sourcing, culture conditions, and MSC-EV isolation methods, is essential to maintain product consistency [15,49]. Stability testing is another critical aspect, as MSC-EVs must demonstrate efficacy and safety over their intended shelf life and storage conditions [47]. Additionally, long-term patient outcome studies are necessary to evaluate the durability of treatment benefits and monitor potential late-onset side effects [47]. Challenges in this regulatory landscape include navigating varying international standards, the need for extensive funding and resources for compliance, and the potential for delays in clinical trials due to stringent review processes [47,49]. Establishing a collaborative framework between researchers, manufacturers, and regulatory bodies can help streamline these challenges and facilitate the successful transition of MSC-EVs into clinical practice [47,49].

Another consideration is the challenges in the storage and stability of MSC-EVs [15]. These EVs are sensitive to environmental conditions, possibly affecting their therapeutic potency over time [15]. Regulatory pathways for MSC-EV therapies are still evolving, and navigating the complexities of approval processes can delay their widespread adoption [15]. Despite these limitations, the results obtained in ongoing clinical studies are continually shedding light on the efficacy of MSC-EVs in clinical settings [47]. Accordingly, innovative strategies are being implemented to support and enhance the beneficial effects of MSC-EVs in the treatment of PD and other neurodegenerative and neuroinflammatory diseases [48,50].

## 6. Future Perspectives for MSC-EVs-Based Therapy of PD

Several approaches could be used for the enhancement of immunosuppressive and neuroprotective properties of MSC-EVs [22,23,48]. Priming MSCs with inflammatory cytokines, TNF-α and IL-1β, can enhance their immunosuppressive properties by up-regulating the production of immunosuppressive factors (IDO, PGE2, IL-10, and TGF-β) [22,23]. Accordingly, EVs derived from TNF-α and IL-1β-primed MSCs contain elevated concentrations of these immunosuppressive proteins, enabling more efficient attenuation of immune cell-driven neuroinflammation [22,23]. Similarly, MSCs that were primed with α-SN have been shown to secrete increased levels of neurotrophic factors (BDNF and GDNF) [48]. Accordingly, EVs obtained from α-SN-primed MSCs were able to more efficiently support the survival of dopaminergic neurons in mice with PD than EVs that were derived from α-SN-non-primed MSCs [48]. It is highly expected that a combination of these two priming strategies (priming with inflammatory cytokines and α-SN) could result in the derivation of MSC-EVs capable of effectively targeting both the immunological and neurodegenerative aspects of PD, offering a dual approach for a more efficient MSC-EVs-based therapy that would successfully suppress harmful immune responses and foster neuronal resilience and regeneration [22,23,48].

Although MSC-EVs have the ability to cross the BBB, a significant amount of intravenously infused MSC-EVs may accumulate in the spleen and livers of MSC-EVs-treated animals [22,23,24,25]. Utilizing engineering technology, MSC-EVs can be designed to express receptors that enhance their brain-targeting ability, allowing them to significantly accumulate in inflamed brains, where they can effectively reduce harmful immune responses and attenuate the progression of neuroinflammatory and neurodegenerative disorders [50].

## 7. Conclusions

In summary, MSC-EVs possess significant therapeutic potential for the treatment of PD [22,23,24,25,26,27]. Their unique composition, enrichment in bioactive molecules, and their ability to cross the BBB present a compelling advantage for targeted therapy of neurodegenerative and neuroinflammatory diseases, including PD [22,23,24,25]. By delivering neuroprotective factors, immunosuppressive proteins, and miRNA to injured neurons and brain-infiltrating inflammatory immune cells, MSC-EVs efficiently promote the survival of injured neurons, restore dopamine production, and alleviate neuroinflammation in the SNpc [22,23,24,25,26,27]. Numerous experimental studies have demonstrated that MSC-EVs can suppress the activation of inflammatory immune cells while enhancing the viability and function of dopamine-producing neurons, alleviating PD-related motor and behavioral deficits in animal models [28,29,30,31,32,33,34,35,36]. The results obtained from these preclinical trials indicate that the administration of MSC-EVs represent a promising and innovative therapeutic approach capable of efficiently alleviating symptoms and slowing disease progression in patients suffering from PD. However, it must be emphasized that these findings require confirmation in upcoming clinical trials before MSC-EVs can be considered as a new therapeutic agent for the treatment of PD.

## Figures and Tables

**Table 1:** Proteins involved in the development and progression of PD and MSC-derived factors responsible for the beneficial effects of MSC-EVs in the attenuation of PD.

Molecule(s)	Cell Source	Effect(s)	Ref. No.
Altered LRRK2 kinase	Dopaminergic neurons	Impaired release of dopamine	[3,4]
Altered SNCA protein	Dopaminergic neurons	Increased aggregation of αSN	[3,4]
Altered PARK7 protein	Dopaminergic neurons	Mitochondrial dysfunction	[4,5]
Altered PINK1 kinase	Dopaminergic neurons	Disruption of mitochondrial homeostasis	[4,5]
Altered PRKN ligase	Dopaminergic neurons	Impaired proteasome-dependent degradation of altered proteins	[4,5]
TNF-α, IL-1β	Microglia	Increased expression of E and P selectins on ECs; increased permeability of BBB	[7]
IFN-γ, IL-17	Th1, Th17 cells	Activation of microglia and brain-infiltrated neutrophils; progression of neuroinflammation	[9]
CD9, CD63, CD81	MSCs	Increased uptake of MSC-EVs by target cells	[22]
IL-10, TGF-β	MSCs	Induction of tolerogenic phenotype in DCs; alternative activation of macrophages	[22,23]
IL-1Ra	MSCs	Suppression of IL-1β-driven neuroinflammation	[22,23]
IDO	MSCs	Increased generation and expansion of immunosuppressive Tregs	[23]
HGF	MSCs	Suppression of microglia	[23,24]
miR-133b, miR-21, miR-124	MSCs	Promote resilience of dopaminergic neurons	[23,24]
BDNF, NGF, GDNF	MSCs	Support growth and survival of dopaminergic neurons	[23,24,25]
SOD, catalase	MSCs	Attenuation of oxidative stress-induced injury of dopaminergic neurons	[24,25]
TSG-6	MSCs	Suppression of oxidative stress and attenuation of neurotoxicity	[37]

Abbreviations: synuclein alpha (SNCA); leucine-rich repeat kinase 2 (LRRK2); parkinsonism associated deglycase (PARK7); α-synuclein (αSN); phosphatase and tensin-homolog (PTEN)-induced kinase 1 (PINK1); parkin RBR E3 ubiquitin protein ligase (PRKN); tumor necrosis factor-alpha (TNF-α); interleukin (IL)-1β; endothelial cell (EC); blood-brain barrier (BBB); interferon gamma (IFN-γ); mesenchymal stem cells (MSCs); mesenchymal stem cell-derived extracellular vesicles (MSC-EVs); transforming growth factor beta (TGF-β); interleukin 1 receptor antagonist (IL-1Ra}; indoleamine 2,3-dioxygenase (IDO); T regulatory cells (Tregs); hepatocyte growth factor (HGF); micro RNA (miR); brain-derived neurotrophic factor (BDNF), nerve growth factor (NGF); glial cell line-derived neurotrophic factor (GDNF); superoxide dismutase (SOD); TNF-stimulated gene-6 (TSG-6).

**Table 2:** Molecular mechanisms and beneficial effects of MSC-EVs-dependent modulation of miRNAs and circRNAs activities in dopaminergic neurons and brain-infiltrated immune cells of PD animals.

MSC-EVs-Dependent Effects	Mechanism(s) of Action	Effect(s)	Ref. No.
Targeted delivery of miR-133b, miR-21, and miR-124 to dopaminergic neurons	MiR-133b, miR-21, and miR-124 inhibit apoptosis and improve survival of injured neurons	Restoration of motor functions in MSC-EVs-treated PD rats	[22,23,24]
Enhanced expression of miR-100-5p in dopaminergic neurons	MiR-100-5p attenuates NOX4-driven oxidative stress	Improved mitochondrial function; alleviated neuroinflammation; reduced apoptosis of dopaminergic neurons	[38]
Up-regulated expression of miR-23b-3p in dopaminergic neurons	MiR-23b-3p activates Wnt/β-catenin signaling pathway and reduces oxidative stress	Increased removal of dysfunctional mitochondria and improved survival of dopaminergic neurons	[39]
Targeted delivery of miR-188-3p to microglia and dopaminergic neurons	MiR-188-3p attenuates activation of NLRP3 inflammasome in microglia and inhibits CDK5-dependent pyroptosis of dopaminergic neurons	Alleviated microglia-driven neuroinflammation and reduced degeneration of dopaminergic neurons	[40]
Enhanced expression of miR-34b in dopaminergic neurons	MiR-34b targets the 3′-UTR of α-SN mRNA and decreases synthesis of α-SN protein	Restoration of motor functions in MSC-EVs-treated PD rats	[41]
Down-regulated expression of circRNA.2837 in dopaminergic neurons	Attenuates aggregation of α-SN protein	Reduced bradykinesia and postural instability in MSC-EVs-treated PD rats	[41]

Abbreviations: mesenchymal stem cell-derived extracellular vesicles (MSC-EVs); microRNA (miR); nicotinamide adenine dinucleotide phosphate hydrogen oxidase 4 (Nox4); Wingless/β-catenin (Wnt/β-catenin); Nacht leucine-rich repeat protein 3 (NLRP3); cell division protein kinase 5 (CDK5); alpha-synuclein (α-SN) messenger RNA (mRNA); circular RNAs (circRNAs).

## Data Availability

No new data were created or analyzed in this study.
